# Retinal pigment epithelium atrophy following indocyanine green dye–assisted surgery for serous macular detachment

**DOI:** 10.4103/0301-4738.53069

**Published:** 2009

**Authors:** Vinod Kumar, Basudeb Ghosh, Meenakshi Thakar, Neha Goel

**Affiliations:** Guru Nanak Eye Centre, Maulana Azad Medical College, New Delhi, India

Dear Editor,

We read with interest the article 'Retinal pigment epithelial atrophy following indocyanine green dye–assisted surgery for serous macular detachment' by Hussain *et al.*[1] It is indeed interesting to note the subretinal migration of indocyanine green dye (ICG) dye without any clinical and optical coherence tomographic evidence of macular hole.

When fluid-air exchange is done during vitreo-retinal surgery, as the air fills the vitreous cavity, the fluid collects at the posterior pole which can be aspirated out. Fluid, if present in the subretinal space requires a retinotomy to aspirate it. The authors mention in the case report that the macula flattened following fluid-air exchange. Whether a retinotomy was made in this case, or the fluid was aspirated from the foveal area is not clear from the article. Flattening of macula without making a retinotomy, points towards the presence of a hole.

Secondly, the rationale of silicon oil injection is not very clear in this case. The occurrence of macular pucker after surgery for retinal detachments complicated by severe proliferative vitreo-retinopathy has not shown to be influenced by the choice of intraocular tamponade in the Silicon Oil Study.[2] In fact, a second surgery could have been avoided by using a long-acting gas instead. Also, C3F8 gas has been found to be a more effective tamponade than silicone oil with respect to achieving initial closure of macular holes in a study by Lai *et al*.[3]
